# Direct Effects of Mifepristone on Mice Embryogenesis: An In Vitro Evaluation by Single-Embryo RNA Sequencing Analysis

**DOI:** 10.3390/biomedicines11030907

**Published:** 2023-03-15

**Authors:** Yu-Ting Su, Jia-Shing Chen, Kuo-Chung Lan, Yung-Kuo Lee, Tian-Huei Chu, Yu-Cheng Ho, Cheng-Chun Wu, Fu-Jen Huang

**Affiliations:** 1 Department of Obstetrics and Gynecology, Kaohsiung Chang Gung Memorial Hospital and Chang Gung University College of Medicine, Kaohsiung City 83301, Taiwan; 2 School of Medicine for International Students, College of Medicine, I-Shou University, Kaohsiung City 82425, Taiwan; 3 Center for Menopause and Reproductive Medicine Research, Kaohsiung Chang Gung Memorial Hospital, Chang Gung University College of Medicine, Kaohsiung City 83301, Taiwan; 4 Medical Laboratory, Medical Education and Research Center, Kaohsiung Armed Forces General Hospital, Kaohsiung City 80284, Taiwan; 5 School of Medicine, College of Medicine, I-Shou University, Kaohsiung City 82425, Taiwan; 6 An-An Women and Children Clinic & ART Center, Kaohsiung City 80759, Taiwan

**Keywords:** mifepristone, RU-486, abortion, RNAseq, embryo, GSEA

## Abstract

The clinical use of mifepristone for medical abortions has been established in 1987 in France and since 2000 in the United States. Mifepristone has a limited medical period that lasts <9 weeks of gestation, and the incidence of mifepristone treatment failure increases with gestation time. Mifepristone functions as an antagonist for progesterone and glucocorticoid receptors. Studies have confirmed that mifepristone treatments can directly contribute to endometrium disability by interfering with the endometrial receptivity of the embryo, thus causing decidual endometrial degeneration. However, whether mifepristone efficacy directly affects embryo survival and growth is still an open question. Some women choose to continue their pregnancy after mifepristone treatment fails, and some women express regret and seek medically unapproved mifepristone antagonization with high doses of progesterone. These unapproved treatments raise the potential risk of embryonic fatality and developmental anomalies. Accordingly, in the present study, we collected mouse blastocysts ex vivo and treated implanted blastocysts with mifepristone for 24 h. The embryos were further cultured to day 8 in vitro to finish their growth in the early somite stage, and the embryos were then collected for RNA sequencing (control *n* = 3, mifepristone *n* = 3). When we performed a gene set enrichment analysis, our data indicated that mifepristone treatment considerably altered the cellular pathways of embryos in terms of viability, proliferation, and development. The data indicated that mifepristone was involved in hallmark gene sets of protein secretion, mTORC1, fatty acid metabolism, IL-2-STAT5 signaling, adipogenesis, peroxisome, glycolysis, E2F targets, and heme metabolism. The data further revealed that mifepristone interfered with normal embryonic development. In sum, our data suggest that continuing a pregnancy after mifepristone treatment fails is inappropriate and infeasible. The results of our study reveal a high risk of fetus fatality and developmental problems when pregnancies are continued after mifepristone treatment fails.

## 1. Introduction

Mifepristone (RU-486) is typically used in combination with misoprostol to induce abortion [1]. Mifepristone primarily functions as an antiprogestogen by blocking the effects of progesterone (IC50 = 0.025 nM for the progesterone receptor, IC50 = 2.2 nM for the glucocorticoid receptor, IC50 = 10 nM for the androgen receptor) [2]. In medical abortions, mifepristone blockades of progesterone receptors directly lead to decidual endometrial degeneration, cervical softening, and dilatation, the release of endogenous prostaglandins, and an increase in the myometrium sensitivity to the contractile effects of prostaglandins [3]. A mifepristone-induced decidual breakdown indirectly leads to trophoblast detachment; this causes a decrease in syncytiotrophoblast production of hCG, which in turn results in the decreased production of progesterone by the corpus luteum [4]. Although the mechanisms of mifepristone’s effects on endometrial receptivity are well established, the direct effects of RU-486 on embryos remain elusive.

Remarkably, treatment failure arising from gestational duration increases 10-fold from 0.3% at less than 49 days of gestation to approximately 3% at 64 to 70 days of gestation [1,5]. Although most women whose pregnancies continue after treatment opts for further treatment, such as surgical aspiration, some decide to continue the pregnancy. Recent UK data revealed that among 2673 women who had a medical abortion after 9 to 10 weeks of gestation, 90 women had ongoing pregnancies after treatment, and nine of those women (10%) continued their pregnancies [6]. Thus, even after they undergo an abortion, some women change their minds. However, whether mifepristone directly affects embryogenesis is still unknown; thus, the mifepristone-induced aftereffect of missed miscarriages is also an unanswered question. Therefore, characterizing the direct effectiveness of mifepristone on developing embryos is necessary to better understand the interactions between mifepristone and abortion procedures and the role it plays in missed miscarriages.

Additionally, the nonmedical terms abortion reversal, medical abortion reversal, and abortion pill reversal have been used to describe a purported treatment first reported in a case series in December 2012 [7]. Conceptually, the goal of progesterone proponents is to induce mifepristone antagonization through the use of high doses of progesterone; two small case reports and one large case series have been published on this treatment [8,9]. However, commentaries have described the numerous scientific and ethical problems related to these reports, including a lack of control groups, a lack of confirmation of mifepristone ingestion, failure to establish viability prior to progesterone treatment, and failure to obtain patient consent or institutional review board approval before the provision of experimental treatments to patients [10,11]. Some members of the reproductive rights community may argue that mifepristone antagonization is conceptually impossible and could harm patients. Consequently, before researchers can examine the feasibility of these alleged abortion reversal procedures, mifepristone’s direct impacts on embryogenesis, independent of its effects on embryo implantation and endometrium, must be evaluated.

## 2. Material and Methods

### 2.1. Collection of Mouse Blastocysts

All animals received humane animal care as outlined in the Guidelines for Care and Use of Experimental Animals (Canadian Council on Animal Care, Ottawa, ON, Canada, 1984). Female ICR mice, 6–8 weeks old (National Laboratory Animal Center, Taiwan), were induced to superovulate by injecting 5 IU pregnant mares’ serum gonadotropin intraperitoneally (PMSG; Sigma Chemical Co., St. Louis, MO, USA) followed 48 h later by injection of 5 IU human chorionic gonadotropin intraperitoneally (HCG; Sigma, St. Louis, MO, USA). They were then mated overnight with a single fertile male of the same strain [12]. Mating will be confirmed by the presence of a copulatory plug the following day. ICR female mice and male mice will be kept under a 12-h day, 12-h night regimen, with food and water available ad libitum. The day a vaginal plug was found was defined as day 0 of pregnancy. Blastocysts will be obtained on the afternoon of day 3 of gestation.

### 2.2. Standard Blastocyst Culture

For assessment of implantation in vitro and further embryonic growth, blastocysts were cultured according to the methods described in a previous study (control *n* = 3, mifepristone *n* = 3) [12]. Briefly, The study involved incubating embryos under specific conditions, which included a constant temperature of 37 °C and an atmosphere of 5% CO_2_ and 95% air. The embryos were cultured in a specific medium called CMRL 1066, which was supplemented with glutamine, sodium pyruvate, 50 IU/mL penicillin (Gibco Life Technologies, Grand Island, NY, USA), mg/mL streptomycin (Gibco), and 20% fetal calf serum (Gibco). The blastocysts were initially cultured without any medium replacement for the first two days and then with fresh medium renewed daily until the eighth day. The embryos were inspected daily under a dissecting microscope and classified according to the Witschi method. The criteria used to analyze the data were designed to decrease observer bias. The morphology of embryos following the day-8 culture has been demonstrated in a previous study [12].

### 2.3. Blastocyst Culture and RU-486 Treatment

For RU-486 treatment, 0 and 20 μM RU-486 at the implanted blastocyst stage, which have been cultured from the blastocysts for 2 days, will be allocated into the experimental groups. In the treatment groups, 20 μM of RU-486 will be added to the following 1-day culture. In the remaining 5 days of culture, the culture medium was changed to the original medium with different additives following the protocol of the 8-day culture as previously described in both the treatment and control groups.

### 2.4. Single Embryo RNA Isolation, cDNA Synthesis, and RNA Library Preparation

The samples were lysis, and the double-strand cDNA will be constructed by using SMART-Seq^®^ v4 Ultra^®^ Low Input RNA Kit for Sequencing (Takara Bio USA, Inc., Mountain View, CA, USA). Library preparation was performed with the NEBNext Ultra Ⅱ DNA Library Prep kit for Illumina (New England Biolabs, Beverly, MA, USA). In brief, cDNA was mechanically sheared, followed by reactions of end-repairing, size-selection, 3′A-tailing, and adaptor-ligation to generate indexed libraries. The library is then ready for amplification and size selection by Purification Module with Agencourt AMPure XP Beads (Beckman Coulter, Brea, CA, USA). The qualified libraries were analyzed by FRAGMENT ANALYZER™ Automated CE System (Advanced Analytical Technologies, Ankeny, IA, USA) and quantified by Qubit Fluorometer (ThermoFisher, Waltham, MA, USA).

### 2.5. Sequencing and Analysis

The libraries were sequenced to depths of up to 30 million single-end 75 nt length reads per sample using the Illumina NextSeq 500/550 High Output v2 kit (75 cycles) on an Illumina NextSeq 500 Sequencing System. This analysis pipeline is developed by the CLC Genomics workbench v20.0.3 software package. After removing the adaptor, rRNA, and low-quality sequences, the remaining reads were aligned onto the reference sequence of the mouse genome (GRCm38/mm10) using the TopHat2 splice-junction mapper and calculated expression value (reads per kilobase million/RPKM) of each gene. For the following statistic test and differential expression analysis, we used DEseq2 provided by iDEP.93 web application [http://bioinformatics.sdstate.edu/idep/ (accessed on 30 June 2021)]. to identify differentially expressed genes (DEG) between groups.

### 2.6. Gene Sets Enrichment Analysis

The RNA-seq data generated from control and RU-486 treated embryos were processed normalization to obtain the relative gene expression profiles, and all RNA expression files were analyzed by Gene sets enrichment analysis (GSEA). GSEA was performed using the GSEA v4.0.3 software. For this analysis, the hallmark gene sets from the Molecular Signatures Database-MSigDB were used to perform GSEA, and gene set permutations were conducted 1000 times for the analysis. Enrichment score (ES), nominal *p*-value (NOM *p*-value), and False discovery rate (FDR) value were used to sort enriched significant pathways.

## 3. Results

To examine the direct effect of mifepristone in embryogenesis, we collected the embryos at the blastocyst stage from pregnant mice and cultured the embryos in vitro. After 48 h, we treated mifepristone to the medium for 24 h. Then, we replaced the medium without mifepristone, allowing the cultured embryo to grow and develop for extra five days until the stage of the early somite. Finally, we collected every single embryo for RNA isolation and RNAseq (control *n* = 3, mifepristone *n* = 3).

We sequenced cDNA libraries of embryonic mouse samples from three mifepristone-treated groups and three normal control groups. After filtering the ribosome transcripts with transcriptome data software (TopHat2) and a short-read mapping tool (bowtie), we acquired 28,888,816 to 32,359,487 paired-end reads per sample. Unmapped reads or reads matched to multiple positions (9.24–13.49%) were excluded from further analyses. Consequently, 24,374 mRNA transcripts were detected in the six embryonic samples.

**Figure 1:**
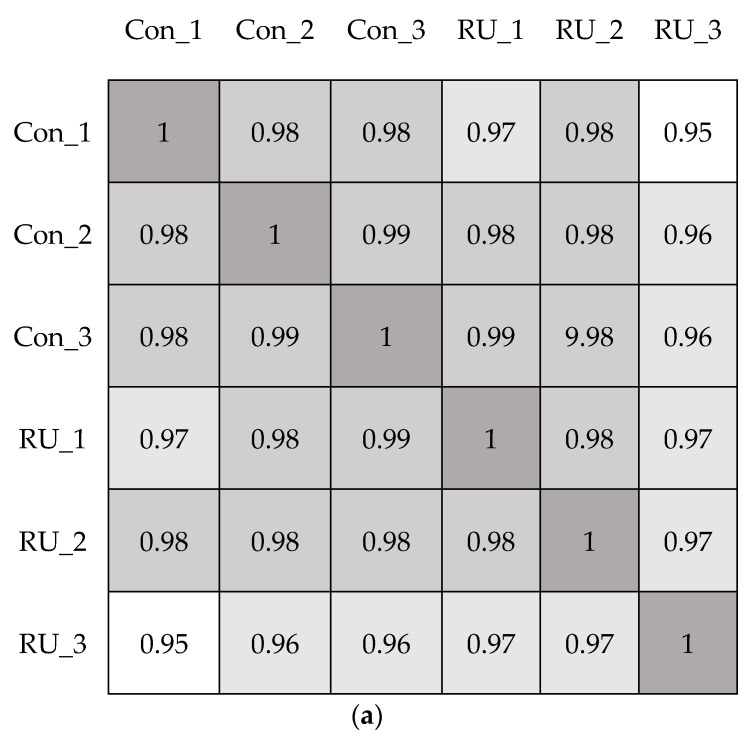

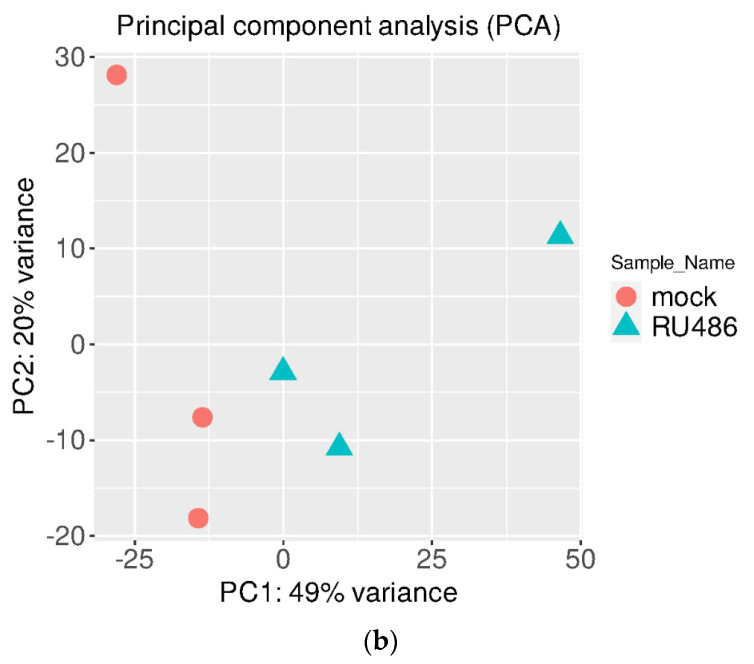
The correlation of RNAseq results. (**a**). Analysis of correlation between mock (con) and RU-486 (RU) treatment embryos by using Pearson correlation coefficient. When R^2^ > 0.95, the two samples were considered correlated. (**b**). Principal-component analysis (PCA) was conducted in the transcriptome of two different treated sample groups. The orange circle indicated the RU-486-treated group, and the blue triangle represented the control group.

After using a pairwise approach, we used three embryos in the same group to eliminate the background noise caused by transcriptions unique to individuals, thus allowing us to acquire more relevant data from the two groups. Further RNAseq analysis was conducted by using iDEP web software [http://bioinformatics.sdstate.edu/idep/ (accessed on 30 June 2021)]. The Pearson correlation coefficients (R2) between every sample pair were calculated based on FPKM (Fragments Per Kilobase per Million); values were obtained as shown in Figure 1a. In addition, the principal component analysis (PCA) was also performed in Figure 1b. The results showed high correlation and similarity among the control and RU-486 samples, and it suggests that the RU-486 treatment may affect the expression of a small subset of genes.

**Figure 2:**
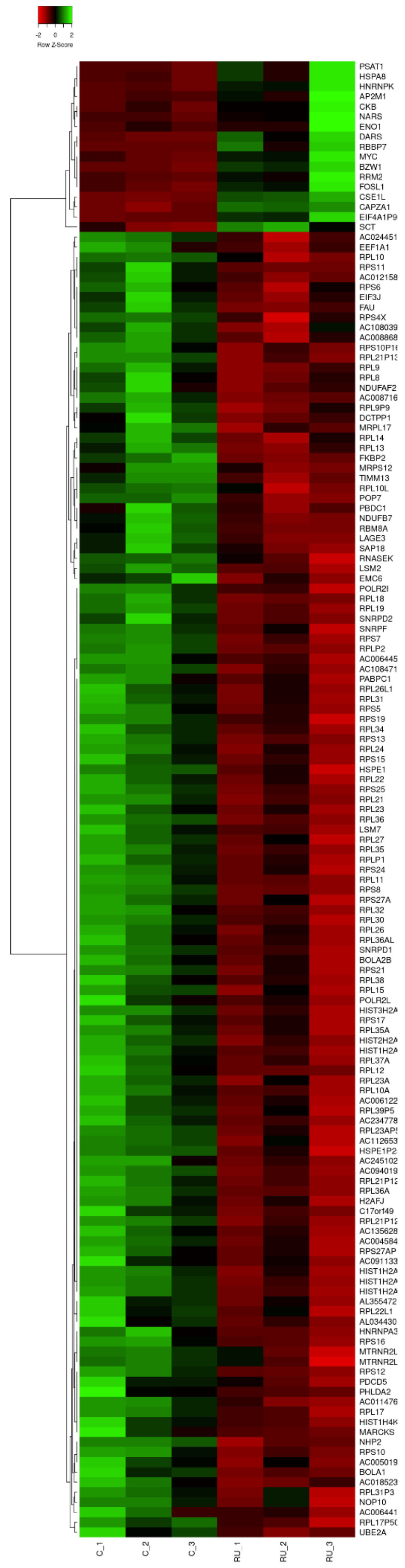
The clustering heatmap indicated the patterns of 147 differential expression genes among the mock and RU-486-treated samples.

To identify the differential expression genes in embryos treated with RU-486, we normalized the RNAseq data by the DEseq2 program. Notably, we found 147 differentially expressed genes (false discovery rate (FDR) *q* < 0.05 and |log2 FC| > 0.58) in both groups. Among these, 130 were downregulated, and 17 were upregulated (FDR *q* < 0.05 and |log2 FC| > 0.58), and the gene expression patterns are also presented in Figure 2. Details of the differentially expressed genes, their full names, and *q* values are shown in Supplementary Table S1.

**Figure 3:**
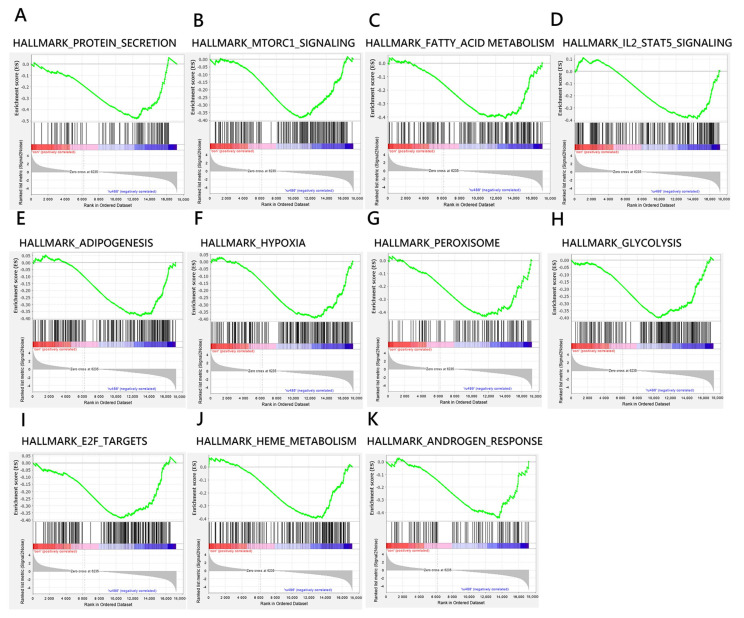
Enrichment plots indicated significantly enriched gene sets (FDR *q*-value < 0.25; NOM *p*-value < 0.05) for embryos treated with RU-486 compared with mock control. (**A**–**K**) show negative enriched score (ES) where the leading edge subset appears in the valley score of the subsequent ranked list.

To characterize the effects of mifepristone, we used gene set enrichment analysis to evaluate gene profiles (Figure 3). Mifepristone treatment considerably altered the cellular pathways of embryos, including important functions of the embryonic cellular process of mice, such as cell viability, proliferation, and development. The data indicated that mifepristone was involved in hallmark gene sets of protein secretion (NOM, abbreviation of nominal, *p* = 0.004), mammalian target of rapamycin complex 1 (mTORC1) regulation (NOM *p* = 0.02), fatty acid metabolism (NOM *p* = 0.008), IL-2 STAT5 signaling (NOM *p* = 0.012), adipogenesis (NOM *p* = 0.018), peroxisome function (NOM *p* = 0.013), glycolysis (NOM *p* = 0.003), E2f targeting (NOM *p* = 0.016), and heme metabolism (NOM *p* = 0.011) (Table 1). These data further revealed that mifepristone might interfere with normal embryonic development. The analysis results suggest that continuing a pregnancy after mifepristone treatment fails is infeasible; it incurs high risks of fetus fatality and developmental problems.

Mifepristone functions as a glucocorticoid inhibitor for endometrial receptivity and embryonic implantation; however, whether it directly interferes with embryogenesis through interference with intracellular androgen signaling remains unclear. In the present study, we used a gene set enrichment analysis (GSEA) to determine that the pathway of androgen response (NOM *p* = 0.014) was enriched in mifepristone-treated embryos, which suggests that mifepristone may directly interfere with embryogenesis in addition to endometrial receptivity and embryonic implantation.

## 4. Discussion

The biological mechanism behind mifepristone’s ability to manipulate the uterus microenvironment and induce fetus abortion has been well studied. However, whether mifepristone directly affects embryo cells remains unclear. Therefore, we performed a systemic analysis to evaluate the potential risks of anomalies in fetal development in abortion reversal procedures. In the present study, we used RNA sequencing and a GSEA to identify several cellular pathways enriched in mifepristone-treated embryos because mifepristone treatment may alter embryo development and result in fetal anomalies. The function survey is described hereafter.

We found that embryonic cellular processes of protein secretion were altered by mifepristone treatment. Another study reported that protein secretion competence was correlated with embryonic viability and chromosomal ploidy in the blastocoel [13]. For example, the highly conserved SNARE protein SEC22B mediates diverse critical functions, including phagocytosis, cell growth, autophagy, and protein secretion during embryogenesis; loss of SEC22B function leads to embryonic fatality [14]. Thus, the proposed proteomic-based strategy is involved in the application of clinically relevant biomarkers of embryo quality. Furthermore, the embryonic protein secretion pathway is essential for the production of extracellular signal molecules. Dysfunction in endocytic pathways, posttranslational modification, and membrane dynamics cause patterning defects in embryogenesis and tissue morphogenesis in mammals [15]. Gene encoding and protein translation are temporally and spatially regulated during embryogenesis [16], and any dysregulation could lead to abnormal morphogenesis or to dysplasia.

Through our GSEA, we further validated the dysfunction of the cell growth regulator mTORC1 involved in mifepristone insults. Notably, another study demonstrated that mTORC1 functions as a cell growth regulator and inactivation of mTORC1 during embryogenesis might cause either defect in myogenesis or perinatal death [17]. mTOR affected cell size and proliferation in early mouse embryos and in embryonic stem cells [18]. Indeed, mTORC1 and mTORC2 play essential developmental roles from fertilization to birth; in lung morphogenesis, mTORC1 is associated with the formation of airways and vascular branches [19]. Additionally, mTOR functions as a key regulator of cell quiescence, pluripotency, differentiation, skin development, and cardiovascular development; overall, mTOR is involved in developmental coordination [20,21,22]. Furthermore, the inactivation of mTOR and its substrate, S6 kinase, results in reduced cell size and embryonic fatality. For instance, mouse embryos with mTOR mutations died shortly after implantation due to impaired embryonic and extraembryonic cell proliferation. The homozygous blastocysts appeared normal, but their inner cell masses and trophoblasts failed to proliferate in vitro [18]. Therefore, mTOR deregulation directly affects embryo developmental malformation.

Fatty acid synthesis is critical to embryonic development: most fatty acid synthase null–mutants and heterozygotes died in utero in one study [23]. Some data have indicated that each developmental stage corresponds to the uptake of different fatty acids [24]; thus, fatty acids play a regulatory role in embryogenesis, affecting not only embryonic metabolism but also oxidative stress, membrane composition, cell signaling events, and gene expression [25]. Additionally, lipogenesis, lipolysis, and the transport and oxidation of fatty acids occur in embryonic cells of all mammalian species; these processes are intrinsic components of energy metabolism [26]. Consequently, during embryogenesis, an embryo’s capacity to use exogenous and/or endogenous lipid reserves is important to its development. Because of the implication that mifepristone is also involved in energy metabolism, we determined that the deregulation of fatty acid metabolism was important for our GSEA.

The alteration of adipogenesis was identified in the present study using GSEA; this implied the presence of aberrant organ development, cellular signaling responses, and epigenetic modifications. Because adipogenesis is a tightly regulated cellular differentiation process, that requires the sequential activation of several transcription factors under the control of a cascade of transcription factors, the deregulation of adipose tissue patterning has lifelong implications [27]. Remarkably, adipocyte development in mouse embryonic stem cells is associated with blood vessel morphogenesis and neural development, and the early stages of adipocyte formation involve major changes in signaling and transcriptional networks that occur on a genome-wide scale [28]. In summary, the alteration of adipogenesis is another consequence of mifepristone treatment during embryogenesis.

Deregulation of the hypoxia cellular process was also identified in our GSEA. A number of oxygen-sensing pathways include the energy and nutrient sensor mTOR, the unfolded protein response, and the nuclear factor (NF)-κB transcriptional response [29]. The transcriptional response mediated by hypoxia-inducible factor (HIF) is also a key component of the cellular response to hypoxia and mammalian embryo development [30]. Accordingly, HIF is an essential intracellular regulator in trophoblast proliferation and differentiation. Additionally, HIF regulates the morphogenesis of the developing heart, and deregulation of HIF leads to the defective development of the myocardium and affects the development of the heart’s vascular endothelium. Furthermore, HIF is required for the migration of neural crest cells [31]. In Hif1-α null embryos, neural crest cells form but do not migrate ventrally into the head mesenchyme and branchial arches [32]. However, hypoxia and HIF are required for chondrogenesis [33], which involves mesenchyme condensation and chondrocyte formation. Deletion of Hif1-α early or late in chondrogenesis leads to massive cell death in proximal limb bones [30,34]. Consequently, hypoxia is a normal and essential part of embryonic development, and the deregulation of hypoxia intracellular signaling is associated with congenital malformation.

In the present study, alteration of peroxisome cellular processes was identified in our GSEA. Under normal physiological conditions, peroxisomes are variable and dynamic cell organelles; their size, shape, number, and protein content can vary greatly across cell types, developmental states, and environments. Additionally, peroxisome dysfunction and alterations may cause epigenetic modifications, such as DNA and histone methylation, histone acetylation, and non-coding RNA regulation [35]. In most organs, the maturation of peroxisomes correlates with the acquisition of specific functions, such as lipid metabolism, thus supporting the role of organelles in tissue differentiation [36]. Accordingly, mifepristone may also affect peroxisome functional modulation and, therefore, embryogenesis.

The bioenergetic and metabolic signaling functions of metabolism can affect multiple aspects of mammalian development [37]. Abnormal glycolysis on a cellular level was confirmed in our GSEA. Notably, mammalian embryos transiently perform aerobic glycolysis through the Warburg effect (WE), a metabolic adaptation also observed in cancer cells [38]. Although this is a comparatively inefficient means of generating ATP, this adaptation allows cells to satisfy other critical metabolic requirements, including biomass production and redox regulation. Many cells use aerobic glycolysis during rapid proliferation, which suggests that this process may support cell growth at a fundamental level [39]. Thus, proliferating cells gain a selective growth advantage through the WE. In one study, the spatiotemporal regulation of glycolysis was observed in the posterior regions of mouse and chicken embryos during embryogenesis; the observed phenomenon confirmed that a gradient of glycolytic activity is associated with FGF and Wnt signaling in the course of elongation of the body axis in amniote embryos [38]. Additionally, another study found that glycolysis is essential for embryonic muscle growth through promotion of myoblast fusion-based muscle growth [40]. In summary, mifepristone treatment may cause defective glycolysis, which is another key abnormal cellular process in mifepristone-induced embryonic developmental malformation.

E2F-bound promoters correspond to transcriptional regulators and genes, such as members of the Notch family, fibroblast growth factors, and Wnt and Tgf-β signaling pathways, that control cell-fate determination [41]. Data from other studies have demonstrated that E2F genes are essential for the formation of ventral and posterior cell-fate determination during early embryogenesis [42] and essential for the promotion of cell cycle-regulated, cyclin-dependent kinase activity during embryonic stem cell differentiation [43,44]. Additionally, E2F3 is essential for normal cardiac development. Loss of E2F3 impairs the proliferative capacity of the embryonic myocardium, and most E2F3-null mouse embryos die in utero or perinatally with hypoplastic ventricular walls and/or severe atrial and ventricular septal defects [45]. However, STAT (signal transducers and activators of transcription) proteins are activated in response to the presence of numerous cytokines, growth factors, and hormones [46]. STAT5a-deficient mice exhibit defective mammary gland development, and STAT5a deficiency alters the sexually dimorphic effects of growth hormones in the liver. STAT5a and 5b also play different biological roles in the immune system [47]. The transcription factor STAT5 is an early marker of the differentiation of murine embryonic stem cells [48]; this differentiation affects the control of embryonic hematopoiesis [49].

A broad range of biological processes involves heme because heme is a key component of many hemoproteins (heme-containing proteins). Heme (as iron-protoporphyrin IX) is an essential cofactor involved in multiple biological processes, such as oxygen transport and storage, electron transfer, drug and steroid metabolism, signal transduction, and micro-RNA processing. However, “excess free-heme is highly toxic due to its ability to promote oxidative stress and lipid peroxidation, thus leading to membrane injury and ultimately apoptosis” [50]. During embryogenesis, heme synthases are expressed in the developmental course of neural tissue and in migrating neural crest cells, which means that heme metabolism may play an important role in neural development [51]. Accordingly, heme modulates gene expression and cell proliferation and differentiation [52,53,54], which implies that it has a potential role in embryonic developmental malformation.

Androgen contributes to male prenatal development, male pubertal development, and spermatogenesis [55,56]. The reduced ability of an XY-karyotype fetus to respond to androgens can further result in infertility and several forms of intersex conditions [57]. Additionally, androgen receptors (AR) are members of the nuclear receptor superfamily that act as a ligand-dependent transcription factor. The receptors play a pivotal role in sexual development and reproduction [58]. Mutations in the AR sequence can cause numerous physiological disorders, such as partial and complete androgen insensitivity syndromes, that lead to abnormal sexual development [59,60]. Additionally, AR mRNA was expressed both in the inner cell mass from blastocysts and in undifferentiated ESCs, implying that the role of AR during ESC differentiation is stage-dependent [61].

Studies on the use of mifepristone alone for pregnancy termination are limited. The largest prospective study of 46 women who continued their pregnancy after mifepristone exposure did not find a significant increase in fetal malformations related to mifepristone exposure [62]. Therefore, based on the summary from evidence-based medicine platforms such as The UpToDate, the current conclusion is that medication abortion during the first trimester is not associated with teratogenic effects. However, as more evidence and related studies become available, current insights may be further advanced.

It is important to note that the safety of medication abortion, including the use of mifepristone and misoprostol, should be carefully evaluated on a case-by-case basis. Potential risks and benefits should be discussed with a healthcare provider, as the risks associated with a medication abortion, including the potential for fetal malformations, can depend on various factors, such as the timing of the medication, dosages used, and the patient’s medical history. Remarkably, mifepristone and misoprostol are often used in combination for medical abortion, and the frequency of this treatment regimen depends on various factors, including gestational age and patient and healthcare provider preferences. However, several lines of evidence indicate that misoprostol exposure can increase the incidence of congenital malformations [63,64,65]. This further emphasizes the potential risk of continuation of pregnancy in cases where the drugs are used in combination.

While current information explains that mifepristone affects the endometrium to disrupt decidual formation, our study is the first to investigate the direct effect of mifepristone on developing embryos. We provide bioinformatics data to explain how it potentially affects embryonic development and the risk of continuation of pregnancy after mifepristone administration. As such, healthcare providers should be aware of the potential risks and help patients carefully consider their options while engaging in complete and informed discussions.

## 5. Conclusions

The gene expression patterns in several cellular processes were determined to be influenced by mifepristone administration in vitro through transcriptome sequencing and GSEA, and we conclude that mifepristone is an essential intervention that may change normal embryonic growth and development in mouse embryos. In summary, ongoing pregnancy following the failure to induce an abortion using mifepristone can potentially increase the risk of developmental malformations and embryonic fatality.

## Figures and Tables

**Table 1:** Gene sets enriched in the transcriptome of RU-486 treated group.

NAME	SIZE	ES	NES	NOM *p*-Value (*p* < 0.05)	FDR *q*-Value (*q* < 0.25)
HALLMARK_PROTEIN_SECRETION	94	−0.481	−1.564	0.004	0.087
HALLMARK_MTORC1_SIGNALING	187	−0.384	−1.344	0.020	0.125
HALLMARK_FATTY_ACID_METABOLISM	155	−0.409	−1.409	0.008	0.126
HALLMARK_IL2_STAT5_SIGNALING	192	−0.389	−1.371	0.012	0.128
HALLMARK_ADIPOGENESIS	194	−0.384	−1.346	0.018	0.134
HALLMARK_HYPOXIA	196	−0.393	−1.376	0.003	0.138
HALLMARK_PEROXISOME	102	−0.435	−1.445	0.013	0.140
HALLMARK_GLYCOLYSIS	193	−0.400	−1.418	0.003	0.141
HALLMARK_E2F_TARGETS	192	−0.387	−1.348	0.016	0.146
HALLMARK_HEME_METABOLISM	184	−0.396	−1.381	0.011	0.150
HALLMARK_ANDROGEN_RESPONSE	95	−0.444	−1.455	0.014	0.179

ES: enrichment score; NES: normalized enrichment score; NOM *p*-value: nominal *p*-value; FDR *q*-value: False discovery rate *q*-value.

## Data Availability

The data used to support the findings of this study are included within the article. Other data that might be useful for the findings of this study will be supplied as supplementary information by the corresponding author (C.C. Wu) upon request.

## Supplementary Materials

The following supporting information can be downloaded at: https://www.mdpi.com/article/10.3390/biomedicines11030907/s1, Table S1: The list of differential expression genes in RU-486 treated embryos.
