# Supplementary material for: Direct Effects of Mifepristone on Mice Embryogenesis: An In Vitro Evaluation by Single-Embryo RNA Sequencing Analysis
Source: Biomedicines. 2023 Mar 15;11(3):907. doi: 10.3390/biomedicines11030907 (PMC10046204; doi:10.3390/biomedicines11030907)
Supplement: Supplementary file 1 [file biomedicines-11-00907-s001.zip › biomedicines-2241029-supplementary.pdf]

Supplementary Table S1. The list of differential expression genes in RU-486 treated embryos.

Regulation	Gene symbol	Accession number	log2 Fold Change	FDR ($p < 0.05$)	Normalized RPKM					
					C_1	C_2	C_3	RU_1	RU_2	RU_2
Up	SCT	ENSG00000070031	1.11	0.03	397.37	210.04	194.98	739.61	836.27	483.58
Up	ENO1	ENSG00000074800	1.11	0.04	306.12	499.74	314.65	524.43	524.74	2025.54
Up	BZW1	ENSG00000082153	0.77	0.02	305.64	301.86	273.42	534.98	516.64	787.07
Up	RBBP7	ENSG00000102054	0.64	0.04	727.04	719.00	719.93	1446.42	961.91	1711.01
Up	HSPA8	ENSG00000109971	0.72	0.04	1171.39	1244.55	1013.09	2155.30	1447.58	3343.64
Up	DARS	ENSG00000115866	0.68	0.04	210.31	189.00	190.07	379.56	283.03	489.19
Up	CAPZA1	ENSG00000116489	1.15	0.00	165.44	128.55	168.08	406.44	396.81	432.88
Up	CSE1L	ENSG00000124207	1.45	0.00	118.89	109.08	120.63	338.69	356.77	460.38
Up	NARS	ENSG00000134440	1.04	0.03	221.81	223.92	146.33	327.20	340.94	863.35
Up	PSAT1	ENSG00000135069	0.92	0.03	177.34	180.06	142.24	348.51	235.37	589.23
Up	MYC	ENSG00000136997	0.76	0.04	219.04	170.46	159.23	306.14	297.21	540.62
Up	AP2M1	ENSG00000161203	0.98	0.03	206.71	255.54	227.92	444.99	307.78	953.47
Up	HNRNPK	ENSG00000165119	0.86	0.02	599.68	679.35	536.26	1090.66	1028.54	1939.37
Up	CKB	ENSG00000166165	0.80	0.04	231.55	314.31	195.85	374.14	389.04	839.26
Up	RRM2	ENSG00000171848	0.76	0.04	186.48	159.94	156.51	279.71	246.75	531.90
Up	FOSL1	ENSG00000175592	0.94	0.02	141.40	119.10	99.61	235.63	217.71	399.85
Up	EIF4A1P9	ENSG00000262953	0.67	0.04	241.62	249.89	258.46	422.61	412.64	616.63
Down	RPL26L1	ENSG00000037241	–0.79	0.04	440.10	376.47	334.10	249.38	309.79	233.50
Down	RPL18	ENSG00000063177	–0.70	0.03	718.02	770.28	666.06	526.52	537.11	525.82

Down	PABPC1	ENSG00000070756	–0.79	0.04	853.05	835.25	625.95	539.17	619.68	427.53
Down	RPL31	ENSG00000071082	–0.80	0.03	642.56	575.15	510.03	379.01	457.90	345.47
Down	UBE2A	ENSG00000077721	–0.93	0.02	719.06	493.56	561.46	403.06	341.54	375.96
Down	RPS5	ENSG00000083845	–0.71	0.04	1396.58	1321.48	1109.87	908.63	1042.69	841.53
Down	NDUFB7	ENSG00000099795	–0.69	0.03	1353.29	1682.81	1508.73	1200.54	1071.06	1090.50
Down	TIMM13	ENSG00000099800	–0.63	0.04	471.11	524.43	524.71	414.66	366.32	396.85
Down	PBDC1	ENSG00000102390	–0.65	0.04	668.89	912.52	790.22	649.97	543.58	611.62
Down	EIF3J	ENSG00000104131	–0.66	0.04	304.48	369.71	292.52	242.75	221.03	270.52
Down	PDCD5	ENSG00000105185	–0.70	0.05	831.56	640.76	639.68	593.47	516.62	442.36
Down	RPS16	ENSG00000105193	–0.67	0.04	705.96	715.26	591.05	526.40	517.09	469.21
Down	POLR2I	ENSG00000105258	–0.83	0.03	506.56	517.64	449.75	360.01	363.17	261.34
Down	RPS19	ENSG00000105372	–0.65	0.05	1438.52	1421.77	1272.55	1092.35	1152.49	878.94
Down	RPL19	ENSG00000108298	–0.61	0.04	1995.54	2095.51	1907.62	1570.16	1617.34	1487.73
Down	RPL34	ENSG00000109475	–0.73	0.04	927.59	814.56	751.17	598.68	662.96	533.99
Down	RPS13	ENSG00000110700	–0.84	0.02	1116.53	1023.23	899.51	657.22	730.81	643.59
Down	RPS12	ENSG00000112306	–0.88	0.02	1137.46	1073.57	931.16	715.46	695.30	636.47
Down	RPL24	ENSG00000114391	–0.80	0.03	1464.70	1390.27	1164.91	844.59	1081.91	814.98
Down	RPS15	ENSG00000115268	–0.82	0.03	732.99	631.31	566.23	428.66	489.81	387.46
Down	HSPE1	ENSG00000115541	–0.67	0.04	2046.38	1977.09	1953.10	1521.56	1663.70	1258.26
Down	RPL22	ENSG00000116251	–0.82	0.04	310.59	276.79	245.02	185.82	219.49	157.14
Down	RPS25	ENSG00000118181	–0.71	0.03	6117.66	5739.44	5222.01	4067.63	4498.11	3927.51
Down	RPL21	ENSG00000122026	–0.74	0.03	646.84	645.12	562.43	426.74	473.47	425.40
Down	RPS10	ENSG00000124614	–0.72	0.03	653.29	646.26	545.00	430.16	475.70	437.41

Down	RPL23	ENSG00000125691	–0.68	0.04	915.71	806.08	721.23	595.42	679.85	545.15
Down	SNRPD2	ENSG00000125743	–0.73	0.03	608.08	736.06	588.48	462.87	483.13	443.88
Down	EMC6	ENSG00000127774	–0.61	0.04	1039.94	1074.05	1244.75	844.44	947.70	830.16
Down	MRPS12	ENSG00000128626	–0.76	0.04	178.58	236.79	236.80	174.67	137.86	145.30
Down	RPL36	ENSG00000130255	–0.82	0.02	637.61	600.98	570.28	400.69	437.31	389.43
Down	LSM7	ENSG00000130332	–0.76	0.04	347.55	308.70	272.58	228.24	233.69	190.59
Down	RPL27	ENSG00000131469	–0.68	0.04	3051.44	2823.91	2631.12	2093.74	2451.68	1766.29
Down	RPL35	ENSG00000136942	–0.73	0.03	4441.67	4278.86	3672.70	2965.55	3234.51	2704.73
Down	RPS6	ENSG00000137154	–0.60	0.04	2098.36	2289.04	1846.24	1666.97	1450.17	1826.05
Down	RPLP1	ENSG00000137818	–0.80	0.03	930.91	826.02	749.66	575.71	657.27	480.88
Down	RPS24	ENSG00000138326	–0.74	0.04	461.92	444.56	383.58	306.08	350.93	257.80
Down	SNRPF	ENSG00000139343	–0.68	0.05	502.34	514.16	454.42	365.24	416.60	302.58
Down	RPS11	ENSG00000142534	–0.62	0.03	5730.39	6617.73	5443.11	4683.89	4565.47	4607.71
Down	RPL11	ENSG00000142676	–0.68	0.03	1145.89	1120.40	960.48	810.72	832.05	765.85
Down	RPS8	ENSG00000142937	–0.89	0.01	1419.36	1392.48	1232.49	888.01	896.99	821.53
Down	RPS27A	ENSG00000143947	–0.69	0.04	968.15	903.97	819.98	644.22	770.73	560.61
Down	RPL32	ENSG00000144713	–0.70	0.04	418.00	409.92	332.56	292.42	300.06	259.85
Down	NHP2	ENSG00000145912	–0.72	0.02	1310.25	1305.08	1251.50	867.85	973.01	966.94
Down	RPL10	ENSG00000147403	–0.80	0.02	737.46	753.61	714.59	610.26	418.22	484.46
Down	FAU	ENSG00000149806	–0.80	0.01	2004.38	2409.01	1950.96	1437.39	1382.05	1569.53
Down	SAP18	ENSG00000150459	–0.63	0.04	794.02	972.95	831.40	743.27	643.22	605.07
Down	RPL30	ENSG00000156482	–0.77	0.03	662.64	641.04	565.11	456.28	480.16	370.15
Down	EEF1A1	ENSG00000156508	–0–.82	0.02	1269.77	1226.36	889.32	827.72	641.69	840.31

Down	MRPL17	ENSG00000158042	–0.63	0.04	524.53	640.28	599.17	411.01	487.02	462.54
Down	RPL8	ENSG00000161016	–0.76	0.02	612.10	762.39	546.47	411.58	436.03	511.53
Down	RPL26	ENSG00000161970	–0.85	0.02	1433.25	1288.51	1087.98	792.07	999.94	716.17
Down	RPL22L1	ENSG00000163584	–0.79	0.04	679.01	490.33	535.61	391.17	482.74	296.82
Down	RPL9	ENSG00000163682	–0.66	0.04	367.24	393.98	333.74	265.94	271.28	296.61
Down	NDUFAF2	ENSG00000164182	–0.83	0.02	306.66	385.06	251.52	187.16	214.62	237.24
Down	RPL10L	ENSG00000165496	–0.74	0.03	2497.42	2593.91	2622.69	2225.77	1498.41	1776.92
Down	RPL36AL	ENSG00000165502	–0.70	0.04	5252.03	4708.51	4007.65	3411.87	3804.05	3024.85
Down	SNRPD1	ENSG00000167088	–0.70	0.04	416.75	399.49	365.89	296.04	312.38	256.69
Down	RPL13	ENSG00000167526	–0.62	0.03	739.99	869.61	825.55	617.41	599.13	679.57
Down	BOLA2B	ENSG00000169627	–0.74	0.04	790.15	707.50	602.94	501.36	567.71	424.46
Down	HNRNPA3	ENSG00000170144	–1.12	0.01	280.08	319.45	200.97	156.28	156.01	124.75
Down	RPS21	ENSG00000171858	–0.77	0.03	1555.63	1542.41	1361.13	995.44	1213.91	895.89
Down	RPS7	ENSG00000171863	–0.78	0.02	783.68	786.41	710.78	519.01	583.76	478.64
Down	POP7	ENSG00000172336	–0.68	0.04	280.50	280.59	292.49	231.46	200.03	206.58
Down	RPL38	ENSG00000172809	–0.79	0.03	1067.86	899.65	767.98	634.08	715.15	540.21
Down	FKBP2	ENSG00000173486	–0.65	0.04	274.54	290.93	310.90	223.10	216.74	227.88
Down	RPL15	ENSG00000174748	–0.69	0.04	1077.89	973.71	953.97	696.89	866.84	658.62
Down	RPLP2	ENSG00000177600	–0.72	0.02	1709.75	1785.34	1611.75	1239.58	1283.95	1167.76
Down	POLR2L	ENSG00000177700	–0.79	0.04	637.60	483.59	420.72	358.56	411.23	294.14
Down	BOLA1	ENSG00000178096	–0.79	0.03	297.72	230.16	238.52	172.33	187.24	173.43
Down	DCTPP1	ENSG00000179958	–0.75	0.03	356.01	499.65	395.23	276.68	292.49	322.47
Down	HIST3H2A	ENSG00000181218	–0.93	0.02	10402.51	10324.05	8805.01	6048.71	7241.70	5072.83

Down	PHLDA2	ENSG00000181649	–1.00	0.02	1869.25	1075.36	1049.67	840.54	829.99	742.03
Down	NOP10	ENSG00000182117	–0.72	0.04	2544.60	2546.34	2168.80	1672.56	2175.49	1373.32
Down	RPS17	ENSG00000182774	–0.76	0.04	683.93	590.27	521.58	427.79	482.30	354.54
Down	RPL35A	ENSG00000182899	–0.75	0.03	1014.72	977.17	843.88	660.37	770.30	567.36
Down	RPL21P132	ENSG00000183911	–0.72	0.02	2497.73	2502.15	2279.31	1673.70	1889.31	1699.57
Down	HIST2H2AC	ENSG00000184260	–0.73	0.03	849.09	774.23	721.49	537.42	621.06	528.15
Down	RPL14	ENSG00000188846	–0.71	0.03	264.12	306.56	265.52	203.37	181.93	231.91
Down	HIST1H2AI	ENSG00000196747	–0.94	0.02	2893.66	2637.64	2120.42	1652.77	1768.21	1324.76
Down	LAGE3	ENSG00000196976	–0.73	0.04	205.54	253.83	226.58	177.46	158.18	156.68
Down	RPL37A	ENSG00000197756	–0.75	0.04	408.03	355.61	311.06	252.76	282.88	224.98
Down	RPL12	ENSG00000197958	–0.64	0.04	841.83	762.74	663.58	586.40	586.42	570.08
Down	RPS4X	ENSG00000198034	–0.71	0.02	1531.50	1549.50	1443.18	1197.56	893.34	1248.01
Down	RPL23A	ENSG00000198242	–0.71	0.04	609.69	553.91	510.47	380.04	476.63	364.51
Down	RPL10A	ENSG00000198755	–0.72	0.04	742.72	692.71	602.27	506.68	528.21	436.67
Down	LSM2	ENSG00000204392	–0.62	0.04	614.41	669.90	629.40	507.35	518.67	457.39
Down	AC108039.1	ENSG00000213486	–0.64	0.03	572.59	645.25	560.65	438.17	406.03	535.61
Down	AC008716.1	ENSG00000213488	–0.71	0.03	446.83	477.11	406.50	312.95	326.78	337.70
Down	RPL17P50	ENSG00000213700	–0.73	0.04	296.43	264.03	282.46	220.45	212.53	168.95
Down	AC005019.1	ENSG00000213875	–0.78	0.04	497.30	398.10	354.38	257.91	352.37	263.48
Down	AC006122.1	ENSG00000213970	–0.84	0.04	364.42	298.74	272.76	207.31	244.89	168.08
Down	RPL39P5	ENSG00000214289	–0.65	0.05	1160.05	1040.40	1002.36	794.90	927.99	695.71
Down	AC234778.1	ENSG00000214925	–0.71	0.04	948.12	845.18	765.50	627.55	675.68	567.75
Down	RPL23AP59	ENSG00000215002	–0.79	0.04	284.43	272.58	255.33	185.93	217.09	153.50

Down	RNASEK	ENSG00000219200	–0.76	0.04	249.43	250.87	261.72	208.58	184.26	139.73
Down	AC112653.1	ENSG00000230249	–0.72	0.04	378.26	368.68	350.56	251.11	316.77	223.52
Down	HSPE1P25	ENSG00000232015	–0.67	0.04	2447.75	2347.00	2307.79	1800.12	2008.33	1497.23
Down	AL355472.3	ENSG00000236358	–0.86	0.04	307.71	226.07	233.28	159.24	204.68	140.98
Down	AC245102.2	ENSG00000236571	–0.99	0.02	302.20	301.86	210.08	159.97	189.30	140.20
Down	AC094019.1	ENSG00000236732	–0.74	0.04	276.99	266.58	254.87	188.69	204.60	175.93
Down	RPL21P121	ENSG00000237382	–0.80	0.03	424.00	386.36	334.45	263.17	283.05	240.55
Down	RPL9P9	ENSG00000237550	–0.63	0.03	1282.62	1454.18	1290.83	962.06	1013.93	1153.89
Down	RPL31P3	ENSG00000237929	–0.79	0.04	722.06	671.86	568.93	416.00	579.42	352.32
Down	RPS10P16	ENSG00000239917	–0.78	0.02	1119.01	1163.28	919.34	687.18	822.00	714.70
Down	RPL36A	ENSG00000241343	–0.78	0.03	245.37	237.26	208.37	163.14	166.51	149.05
Down	AC006445.1	ENSG00000242209	–0.73	0.03	2164.52	2173.54	1767.12	1534.35	1592.25	1240.31
Down	AC012158.1	ENSG00000243024	–0.61	0.04	494.65	560.12	464.88	413.82	379.57	397.57
Down	AC011476.1	ENSG00000243494	–0.82	0.03	470.01	463.01	391.25	340.81	286.03	269.37
Down	H2AFJ	ENSG00000246705	–0.90	0.03	343.56	334.03	267.05	196.04	244.95	160.04
Down	AC008868.1	ENSG00000248288	–0.60	0.04	543.06	575.41	514.59	439.07	387.30	465.72
Down	AC108471.1	ENSG00000249019	–0.76	0.02	2833.67	2911.86	2638.99	1895.14	2200.77	1819.17
Down	AC024451.1	ENSG00000251354	–0.84	0.02	382.70	372.93	330.99	272.92	195.45	264.41
Down	AC018523.1	ENSG00000255074	–0.61	0.04	730.64	679.11	590.12	502.55	519.09	559.15
Down	MTRNR2L10	ENSG00000256045	–0.98	0.03	1137.56	1161.03	969.21	892.62	630.18	411.68
Down	C17orf49	ENSG00000258315	–0.75	0.04	312.37	253.16	239.60	185.31	203.82	184.59
Down	RPL21P12	ENSG00000259535	–0.77	0.02	1916.77	1871.30	1691.29	1245.31	1415.48	1183.31
Down	AC135628.1	ENSG00000260109	–0.81	0.04	274.86	236.58	200.98	162.08	185.59	135.07

Down	AC004584.2	ENSG00000262623	–0.87	0.02	945.00	878.42	766.31	547.95	678.14	457.47
Down	RBM8A	ENSG00000265241	–0.77	0.03	189.91	254.00	217.75	166.39	146.39	149.08
Down	RPL17	ENSG00000265681	–0.81	0.03	299.91	289.09	249.78	206.24	199.84	162.28
Down	AC006441.3	ENSG00000265784	–1.17	0.03	404.74	321.31	175.20	179.52	196.41	94.23
Down	MTRNR2L12	ENSG00000269028	–0.82	0.04	1938.64	2009.47	1736.64	1628.74	1315.04	820.04
Down	RPS27AP19	ENSG00000269228	–0.78	0.04	798.04	704.46	584.42	482.12	560.73	398.35
Down	AC091133.5	ENSG00000270781	–0.75	0.04	1207.31	894.18	819.66	666.85	809.22	597.64
Down	HIST1H4K	ENSG00000273542	–1.24	0.01	348.69	242.08	209.13	151.31	149.77	102.20
Down	HIST1H2AH	ENSG00000274997	–0.87	0.02	594.78	582.02	490.47	339.57	431.17	313.26
Down	HIST1H2AJ	ENSG00000276368	–0.95	0.01	4137.32	3931.60	3434.23	2309.52	2719.83	2057.58
Down	HIST1H2AL	ENSG00000276903	–0.86	0.02	1640.54	1542.64	1381.02	1009.55	1093.42	883.36
Down	MARCKS	ENSG00000277443	–1.09	0.02	334.76	232.36	180.64	147.94	144.51	129.39
Down	AL034430.2	ENSG00000285723	–0.76	0.04	326.06	226.92	251.01	186.59	207.89	174.95

Accession number: ensemble gene accession number; FDR: false discovery rate; RPKM: reads per kilobase million.
